# Variations and voids: the regulation of human cloning around the world

**DOI:** 10.1186/1472-6939-5-9

**Published:** 2004-12-13

**Authors:** Shaun D Pattinson, Timothy Caulfield

**Affiliations:** 1 Sheffield Institute of Biotechnological Law and Ethics (SIBLE), University of Sheffield, UK; 2 Health Law Institute, Faculty of Law and Faculty of Medicine and Dentistry, University of Alberta, Canada

## Abstract

**Background:**

No two countries have adopted identical regulatory measures on cloning. Understanding the complexity of these regulatory variations is essential. It highlights the challenges associated with the regulation of a controversial and rapidly evolving area of science and sheds light on a regulatory framework that can accommodate this reality.

**Methods:**

Using the most reliable information available, we have performed a survey of the regulatory position of thirty countries around the world regarding the creation and use of cloned embryos (see Table 1). We have relied on original and translated legislation, as well as published sources and personal communications. We have examined the regulation of both reproductive cloning (RC) and non-reproductive cloning (NRC).

**Results:**

While most of the countries studied have enacted national legislation, the absence of legislation in seven of these countries should not be equated with the absence of regulation. Senator Morin was not correct in stating that the majority of recent legislation bans both RC and NRC. Recent regulatory moves are united only with regard to the banning of RC. While NRC is not permitted in seventeen of the countries examined, it could be permitted in up to thirteen countries.

**Conclusions:**

There is little consensus on the various approaches to cloning laws and policies, and the regulatory position in many countries remains uncertain.

## Background

"The immense majority of countries who have passed legislation recently do ban both reproductive and therapeutic cloning" *(Senator Morin, The Standing Senate Committee On Social Affairs, Science And Technology, Ottawa, Canada, Wednesday 18 February, 2004)*.

In February 1997 an article was published in *Nature *announcing the birth of what was to become the most famous sheep in history [1]. That sheep, known as Dolly, was the product of asexual reproduction. As the world's media unhesitatingly announced, she was a clone. The prospect of a human clone led to immediate calls for regulatory controls on the technology. There were, however, divisions, particularly when it became apparent that the potential uses of the technique were not limited to reproduction. Other potential uses came one step closer when, in the following year, it was announced that embryonic stem cells had been successfully extracted from non-cloned human embryos [2]. Now, a year after the death of Dolly [3], it is appropriate to review the current regulatory position on the creation and use of cloned embryos around the world [4,5] – particularly considering the public debate that has surrounded the recent cloning experiments in Korea, the granting of the first "research cloning" license in the UK, and the past and impending UN cloning debates.

This paper is concerned with the creation of *functional *embryos, whether by nuclear transfer or embryo splitting. As this suggests, we will use 'embryo' to refer to any human entity considered theoretically capable of implantation and development in the womb. Many regulatory positions distinguish between the creation of a cloned embryo for reproductive purposes and for other purposes. For our purposes, the former will be called reproductive cloning (*RC for short*) and the latter non-reproductive cloning (*NRC for short*). This paper will examine the regulatory position of the thirty countries for which we have been able to obtain reliable information (see Table 1). Where possible, we have relied on copies of the original legislation or of English translations of that legislation. In some situations we have also found it necessary to rely on other published sources [6] and personal communications.

**Table 1:** Summary of regulation*

**Country**	**National legislation and effect, or approach in the absence of national legislation**
Australia	*Prohibition of Human Cloning Act 2002: *Prohibits both RC and NRC
Austria	*Act No.275 of 1st July 1992: *Implicitly prohibits both RC and NRC
Belgium	*Law of 11th May 2003: *Prohibits RC but permits NRC
Canada	*Assisted Human Reproduction Act 2004: *Prohibits both RC and NRC
China	*None *Ministerial regulations prohibit RC and allow NRC
Denmark	*Law No.460 of 1997: *Prohibits both RC and NRC
Finland	*Medical Research Act No.488 of 1999: *Prohibits RC, *but *NRC might not be included within this prohibition
France	*Law of July 2004: *Prohibits both RC and NRC
Germany	*Embryo Protection Act 1990: *Prohibits both RC and NRC
Greece	*Law 3089 of 23rd December 2002: *Prohibits RC, but does not cover NRC
Iceland	*Law No. 55 of 29th of May 1996: *Prohibits both RC and NRC
India	*None *Guidelines reject RC but might allow NRC
Ireland	*None *Constitutional provision might prohibit both NRC and RC
Israel	*Prohibition of Genetic Intervention (Human Cloning and Genetic Manipulation of Reproductive Cells) Law 1999: *Imposes a moratorium on RC, silent on NRC
Italy	*Law of 2003: *Prohibits both RC and NRC
Korea	*Bioethics Law 2003: *Prohibits RC, silent on NRC
Luxembourg	*None*
Mexico	*General Health Law of 7 May 1997: *Prohibits both RC and NRC
Netherlands	*Embryo Act 2002: *Prohibits RC and imposes a moratorium on NRC
New Zealand	*Medicines (Restricted Biotechnical Procedures) Amendment Act 2002: *Prohibits RC, silent on NRC
Norway	*Law No. 100 of 5 December 2003: *Prohibits both RC and NRC
Peru	*General Law No. 26842 of 9 July 1997: *Prohibits both RC and NRC
Portugal	*None *Ratified Convention (discussed below)
Russia	*Law on the Temporary Prohibition of Human Cloning 2002: *Imposes a Moratorium on RC
Spain	*Law 35 of November 1988: *Prohibits both RC and NRC
Sweden	*Law No. 115 of 14th March 1991: *Implicitly prohibits both RC and NRC (with possible gaps)
Switzerland	*Federal Law of 18 December 1998: *Prohibits both RC and NRC
Thailand	*None*
UK	*Human Fertilisation and Embryology Act 1990: *Permits NRC under licence *Human Reproductive Cloning Act 2001: *Prohibits RC
United States	*None *Some states have legislation prohibiting RC or both RC and NRC

* The International Digest of Health Legislation  provides translations of the legislation of Austria (1993, vol 44); Denmark (1997, vol 48); Israel (2000, vol 51), Norway (2003, vol 54), Peru (1998, vol 49), and Switzerland (1999, vol 50, and 2003, vol 54). The Bulletin of Medical Ethics provides translations of the legislation of Finland (Feb 2000, p7) and Germany (Dec 1990, p9). In addition, we have used English translations of the legislation of Greece , the Netherlands , and Sweden (Ministry of Health and Social Affairs, *Swedish Act concerning Use of Gene Technology on Human Beings and Experiments with Fertilised Ova*, 1991).

Understanding the complexity of this "regulatory patch work" is essential [7]. It provides a sense of the vast differences between nations, the issues on which there are different views, and the existing regulatory uncertainties. In addition, it highlights both the challenges associated with the regulation of a controversial and rapidly evolving area of science and the need for a regulatory framework that can accommodate this reality. Finally, it demonstrates that policy makers cannot rely on the existence of a single regulatory trend to inform policy development. Only on the banning of RC (reproductive cloning) do the world's legislatures and policy-makers display anything approximating a single mind. Senator Morin, who is a member of Canadian Senate, was not right to claim that a majority of recent legislation bans both RC and NRC (non-reproductive cloning). Even looking beyond recent legislation, only a narrow majority of the thirty countries studied actually prohibit NRC. What is more, a large minority of countries have yet to enact national legislation.

This paper is divided into sections. The next, section II, will examine the degree of regulatory variation in the countries studied. Section III will ask whether the existence of legislation answers all regulatory questions. Using examples drawn from those countries with legislation, we seek to show how broad interpretative strategies are sometimes required to avoid unintended lacunae. Section IV uses examples to demonstrate the evolving nature of regulatory positions and increasing reliance on legislation. Section V examines the impact of international initiatives in European (ie the European Convention on Human Rights and Biomedicine) and the United Nations. Section VI will explain why the regulatory outcome usuallysays little about the ethical approach adopted by a particular jurisdiction. Section VII is the conclusion.

### Variation between countries

As many commentators have noted, there is great variation in regulatory approaches even within countries that have decided to create relevant laws and policies [4,5,7,8]. No two countries have adopted identical regulatory measures on cloning, though the effect of those adopted in some countries is very similar. There is only one area of regulatory agreement – no jurisdiction has, yet, adopted legislation or guidelines permitting RC. As a result, there are essentially only two regulatory approaches to RC: prohibition or regulatory silence. Regulatory silence usually means that RC is technically legal in the jurisdiction in question, though if it were attempted, there would likely be a rapid regulatory response.

The majority of the countries studied have now enacted national legislation (see Table 1). Only seven have yet to do so. The absence of national legislation in these seven countries should not, however, be taken to amount to an absence of regulation or, in the case of the US, an absence of state legislation. Legislation is just one of many possible regulatory responses. Ireland provides an illustrative example. The Eighth Amendment to the Irish Constitution (which forms Article 40.3.3) states that,

"The State acknowledges the right to life of the unborn and, with due regard to the equal right to life of the mother, guarantees in its laws to respect, and, as far as practicable, by its laws to defend and vindicate that right."

While this provision does not mention cloning, it has been taken to protect *in vitro *embryos and thereby prohibit NRC. In addition, doctors must comply with the guidance of the Medical Council, as this body has the power to remove their licence to practise in Ireland. The Medical Council's guidelines declare that " [t]he creation of new forms of life for experimental purposes or the deliberate and intentional destruction of human life already formed is professional misconduct" [9]. Also, it limits the manipulation of sperm or eggs to the "improvement of health" and adds that "if the intention is...the creation of embryos for experimental purposes, it would be professional misconduct" [9]. Thus, the absence of legislation in Ireland does not render all things permissible.

### Variation and non-reproductive cloning (NRC)

NRC represents the source of considerable regulatory variation. While NRC is not permitted in seventeen of the countries studied, it could be permitted in *up to thirteen *other countries. Regulatory uncertainties make it impossible to be sure in some of these counties. Also, some countries (such as the US) have many jurisdictions, each capable of adopting a different regulatory position. Given the superficial similarity of many of these countries and jurisdictions, it is hard to explain such stark variation on cultural differences alone [8,10].

Only Belgium and the UK have deliberately enacted or extended legislation for the purpose of permitting the creation of cloned embryos for research [6,11]. The UK licensing authority has, in fact, granted its first licence to conduct NRC [12]. Similarly permissive approaches, albeit non-legislative, have been adopted by China (which issued Ministerial Regulations in August 2003 to allow cloning research for therapeutic purposes) [6,13] and Korea (where the government is in the process of approving limited research on limited somatic nuclear transfer research) [6].

In contrast, Finland, Greece, Israel, Russia and Sweden appear to allow NRC only because their legislation has potential gaps [14]. The Greece legislation is the most striking, because it is the most recently enacted. The Greek Law 3089/2002 explicitly prohibits 'human reproduction' by any cloning method, but no mention is made of NRC. This must have been deliberate, because the provision allowing embryo research does so by allowing research (with consent) on "fertilized ova" that are surplus following assisted reproductive treatment. Indeed, the legislation's Explanatory Memorandum declares that

"only reproductive cloning is prohibited. It could be thus construed that therapeutic cloning is permitted....This position has also been supported in the Report of the National Bioethics Committee regarding the use of stem cells in biomedical research and clinical practice (21.10.2001), submitted to the Prime Minister on 11.1.2002."

This is nonetheless controversial in Greece, which has ratified both the European Convention on Human Rights and Biomedicine, and its Additional Protocol on cloning (discussed below).

The US, India, and Portugal are anomalous. In the US, what little national legislation there is only concerns the use of federal funds [4] and some States (such as California and New Jersey) have even adopted permissive legislation [15]. The Indian Council of Medical Research has declared that "research on cloning with intent to produce an identical human being, as of today, is prohibited", but has not declared NRC to be so prohibited [16]. However, an Indian Government policy document "opens the door to therapeutic cloning considered on a case-by-case basis by the National Bioethics Committee" [6]. Portugal has no national legislation, but has ratified the European Convention and its Additional Protocol (see below).

### Legislative gaps and uncertainties

There are, of course, many nations that have long standing laws that are relevant to cloning technologies. In many of these nations, however, the laws were designed prior to Dolly and the recent advances in stem cell research. As such, how these laws might apply to cloning is sometimes unclear. Also, these laws are not a result of a public and political dialogue about the complex scientific and ethical issues that are associated with cloning and stem cell technologies. [10] In addition, there are a number of countries where recent legislative intervention has failed to answer all legal questions relating to human cloning.

The legislation of some countries clearly encompasses somatic cell nuclear transfer (SCNT). The Spanish Law 35 of November 1998 is an example of a pre-Dolly legislation of this type. This Act not only renders it an offence to create identical human beings where it is aimed at race selection, it also renders it an offence to create "human beings by cloning in any of the variants or any other procedure capable of originating several identical human beings." The Canadian Assisted Human Reproduction Act 2004 is an example of post-Dolly legislation of this type. Under this Act, the creation and implantation of a "human clone" are prohibited. "Human clone" is defined under s. 3 to mean "an embryo that, as a result of the manipulation of human reproductive material or an *in vitro *embryo, contains a diploid set of chromosomes obtained from a single – living or deceased – human being, foetus or embryo". This clearly captures SCNT.

In a number of contrasting countries, SCNT is only captured by a broad, non-literal interpretation of the relevant provisions. The Swedish Law No. 115 of 14 March 1991 is an example of pre-Dolly legislation of this type. This Act only regulates experiments performed on "fertilised ova" or gametes "before fertilisation". The Finnish Medical Research Act 1999 is a rare example of post-Dolly legislation of this type. This Act has many provisions with respect to research on embryos (including a prohibition on the creation of embryos for research) and it prohibits all research conducted with the aim of cloning a human being. However, s. 2 of the Act defines an embryo as "a living group of cells resulting from fertilisation not implanted in a woman's body". Thus, the Dolly technique only appears to be covered insofar as its use involves "research with the aim of cloning human beings" (Also, s.1 of the Constitution secures the inviolability of human dignity, but there is no authoritative interpretation on whether (and how) this provision could apply to cloning). If this aim is only correctly attributed to RC, then NRC might not be covered at all.

The dangers of non-literal interpretation of pre-Dolly provisions should not be exaggerated. Although there are a number of countries that have such legislation (notably, Austria and Germany and, until very recently, France) [5], in reality the courts are likely to adopt a broad, purposive approach to interpretation. A very broad approach was, for example, taken when the domestic courts addressed the UK's Human Fertilisation and Embryology Act 1990 [17]. The Act explicitly prohibited only one form of cloning (the creation of a clone by replacing the nucleus of an embryo) leaving the licensing authority to regulate activities such as the creation, storage, and use of *in vitro *embryos. More precisely, the Act imposes a licensing requirement on the creation of an *in vitro *embryo (ss.3(1)(a) and 1(2)); storage or use of *in vitro *embryos (ss.3(1)(b) and 1(2)); storage of gametes (s.4(1)(a)); and use of gametes, unless 'services are provided for the woman and man together' (s.4(1)(b)). Yet, under s. 1(1) of the Act, "embryo" is defined as "a live human embryo where fertilisation is complete", including "an egg in the process of fertilisation". This raised the question of whether SCNT fell outside the Act altogether. Nonetheless, the House of Lords recently held that SCNT produces a functional embryo that falls within the ambit of this Act (in effect, holding that the Act's definition of embryo is non-exhaustive and restricted in purpose) [18].

### Evolving nature of the laws

Not only is there a great deal of variation between nations and much uncertainty as to the scope of existing laws, many of the existing laws and policies are in a state of flux. Indeed, some countries have built in review provisions.

The Dutch legislation, the Embryo Act 2002, presents an example. This Act prohibits procedures undertaken for the purpose of creating genetically identical human individuals and prohibits the creation of embryos for research. Yet, s. 33 of the Act allows for the future repeal of the prohibition on the creation of embryos for research. Likewise, the recently enacted Canadian legislation states that a Parliamentary review of the law is required within three years of proclamation.

Some countries, such as Israel, New Zealand and Russia, have even adopted time-limited legislation. The Israel legislation of 1999, for example, states that, *for a period of 5 years*, no intervention will be carried out on human cells for the purpose of human cloning or to bring about the creation of a person by the use of reproductive cells that have undergone permanent intentional genetic modification [19]. What is more, the Act states that the Minister of Health may (upon satisfaction of a number of conditions) permit the creation of a human being through the use of genetically modified cells. Non-legislative bans are often time-limited or chosen because of the ease with which they can be reconsidered.

Other countries are in the process of considering a revision to their existing law. Ireland has set up a Commission on Assisted Human Reproduction in 2000 to explore this topic [20] and the Irish government has officially stated its opposition to cloning [21]. There are voices calling for revision of the Germany legislation [22]. The recently passed Italian legislation might have to be reconsidered because a referendum on a disputed law can be forced if 500,000 signatures are obtained and it has been reported that over a million people have signed a petition calling for a referendum [23]. The Swedish legislation might well be amended in the near future, to close the gaps mentioned in the last section. If the Government Bill 2003/04:148 on stem cell research is enacted, it will come into force on the 1st of January 2005. This Bill seeks to extend the existing legislation to make it clear that RC using the somatic nuclear transfer is encompassed *and *to explicitly allow somatic nuclear transfer as a way of creating embryos for non-reproductive purposes. Thus, Sweden is likely to join Belgium and the UK in permitting NRC by legislation.

### International initiatives

#### i) European Convention

The European Convention on Human Rights and Biomedicine has now been signed by 31 of the 45 member States of the Council of Europe, of which 15 have also ratified the Convention [24]. It has not been signed by any of the non-member participants (which include Australia, Canada, the Holy See, Mexico, and the US). While this Convention does not specifically address human cloning, a number of its provisions have implications for cloning.

Article 18(2) of the Convention prohibits the "creation of human embryos for research purposes". The phrase "human embryos" is not defined by the Convention and subsequent negotiations of the working party on the protection of the human embryo and fetus appear to have failed to reach agreement on this and other issues. This provision only prohibits NRC if it captures the creation of all *functional *human embryos for research. While the Convention makes provision for referrals of questions of interpretation to the European Court of Human Rights (Article 29), referral is unlikely because the Convention arguably leaves such decisions to the discretion of individual States. The Strasbourg court itself allows individual States a wide discretion (known as the 'margin of appreciation') in controversial policy areas. The court has, for example, adopted this approach when considering whether the fetus is included in the provision of the European Convention on Human Rights and Fundamental Freedoms that grants 'everyone' a right to life (see the latest case: *Vo v France *(no. 53924/00)). Moreover, before signing or ratifying the Convention on Human Rights and Biomedicine, any State could make a reservation to this provision insofar as it is inconsistent with their pre-existing law (Article 36). This is what we would expect the UK to do if it eventually signs the Convention.

Whether RC is implicitly prohibited by the Convention is more controversial. Those who hold that cloning violates human dignity will no doubt point to Article 1, which requires parties to the Convention to "protect the dignity and identity of all human beings". This seems tenuous. There is, however, an Additional Protocol on the Prohibition of Cloning Human Beings [25]. Article 1 of the Additional Protocol declares that,

Any intervention seeking to create a human being genetically identical to another human being, whether living or dead, is prohibited.

Since "genetically identical" is defined, under Article 1(2), as "sharing with another the same nuclear gene set", use of the Dolly technique on humans is included within this prohibition. This provision clearly captures RC. What is more controversial is whether it covers NRC. To foreclose this possibility, when the Dutch government signed the Protocol it added an interpretative statement declaring that it "interprets the term "human beings" as referring exclusively to a human individual, ie a human being who has been born". This interpretative statement is arguably unnecessary, because, in the absence of a definition of human being in the Convention itself, States are free to interpret this provision in accordance with their own national policy.

The impact of these international instruments is particularly important with regard to the three countries that have ratified both: Portugal (which has no legislation), Greece (whose legislation only explicitly prohibits RC), and Spain (which has comprehensive legislation in this area). In Greece, the Explanatory Memorandum to the legislation declares that ' [i]t could be...that therapeutic cloning is permitted exactly as in Article 1 paragraph 1 of the Additional Protocol on Cloning'. We understand that conservative opinion is of the view that this interpretation is in conflict with Article 18(2) of the Convention itself. However, neither the Convention nor the Protocol on Cloning provide any sanctions for violation.

#### ii) United Nations

The United Nations' struggle to agree on a cloning treaty exemplifies both the variation of approaches and the challenges associated with seeking consensus in a morally contested area [26]. In December 2001, the UN General Assembly established an Ad Hoc Committee to consider "the elaboration of an international convention against the reproductive cloning of human beings" [27]. Since that time, a number of treaty proposals have been considered. A proposal by France and Germany, for example, recommended a narrow ban on RC only, leaving NRC for future debate [28]. A second proposal supported by Spain and the US, argued for a comprehensive ban on cloning, including NRC [29]. The most recent proposal, which was put forward by Costa Rica, would require states to establish criminal offences for all human cloning, including NRC [30].

There has, however, been little consensus on how to proceed. Though all countries agree that RC should be banned, there is deep division regarding NRC. Neither the Ad Hoc cloning committee nor the UN's Legal Committee could reach a consensus on which proposal to support and bring before the General Assembly. In November 2003 the Legal Committee voted (80-79) to recommend a two-year deferral on a General Assembly decision – a compromise that was put forward and supported by most of the members of the Organization of the Islamic Conference. This decision was largely seen as a victory for those countries supporting a more permissive approach to cloning policy [31]. Indeed, some viewed the two year delay as an ideal opportunity for the scientific community to promote the value of NRC [32]. However, in response to pressure from those countries seeking a comprehensive ban, the General Assembly came to yet another compromise. In January 2004 the General Assembly overturned the Legal Committee's recommendation and supported a one year delay on the debate over the cloning treaty. This October, the General Assembly re-opened the debate, again with no apparent compromise from either camp [33].

The fact that the deep division at the UN is primarily about NRC reflects the lack of any consistent approach to cloning policy. For example, one would expect an emerging trend toward the banning of NRC, as suggested in the quote by Senator Morin, to be reflected in the building of consensus or, at least, a degree of flexibility at the UN General Assembly.

### Ethical considerations

Few areas of regulation are as evidentially driven by ethical views as the regulation of cloning and cloning research. This is not the place for in-depth analysis of the underlying debate. Elsewhere we have both argued that existing regulatory attempts to prohibit RC have rarely been underpinned by thoughtful exposition of underlying ethical principles [5,10,34]. Policy statements frequently rely on claims that are tautologous, under specified, poorly considered, or a combination of these things. Our claim here is more modest. In this section we seek to show why attempts to understand the ethical basis of the existing law cannot focus solely on the existing regulatory outcomes. And, of course, the regulatory outcome does not, necessarily, represent a jurisdictional consensus on the central ethical issues.

Lawyers rarely look to regulatory outcome to understand ethical debates. Unfortunately, in the area of cloning many commentators do that very thing. As our starting quotation demonstrates, politicians and commentators are all too ready to find support for their ethical views in regulatory positions adopted elsewhere. There are, however, varying levels of ethical agreement. Agreement on the appropriate regulatory position does not imply agreement on the underlying ethical principles.

Consider the relationship between ethical positions on the moral status (or dignity) of the cloned embryo and NRC. The cloned embryo could be considered to have full, no, or limited moral status [18,35]. The *full status *position would grant the embryo the same level of moral duties as you or I. The *no status *position would grant the embryo no more status or dignity than your hair or nails. The *limited status *position would grant the embryo a fixed or gradualist level of intrinsic moral value between these two extremes. The full status position will require the prohibition of NRC (destructive use of embryos is considered murder) and the no status position will usually require NRC to be permitted (unless such an approach will interfere with the moral interests of those who do matter). The limited status position is, however, potentially compatible with either regulatory position, depending on the particular status given to the early embryo and the weight given to potential benefits of NRC. It follows that the fact that the regulatory position permits or prohibits NRC does not do even tell us what status the embryo is considered to have. This is further complicated by the fact that supporters of the no or limited status position might be prepared to accept more restrictions than are strictly required by their position to protect a more important moral goal. Only supporters of the full status position cannot coherently make such pragmatic compromises [18].

Similarly, the existing positions on RC could be supported by radically different ethical views. Prohibitions could be supported by those who hold that RC is absolutely wrong (eg always violates human dignity) and by those who hold that cloning at present would be wrong. Even time-limited prohibition does not enable us to discern whether it is underpinned by, for example, the view that RC is wrong because of current safety issues or the view that RC is not wrong but the most effective way to get there is by initially prohibiting it.

In sum, the majority of regulatory outcomes could be coherently explained by reference to one or more underlying ethical positions. Thus, similar or even identical regulatory outcomes imply less by way of ethical agreement than some may be inclined to believe.

## Conclusion

Cloning laws and policies are far from uniform across the globe and the legal position in some countries remains uncertain. This will give little comfort to scientists and policy makers hoping to gain clear direction from the international position. For the time being at least, policymakers must accept the reality of international "dissensus" and scientists wishing to undertaken research on NRC are best advised to consider conducting their research in only a handful of countries. Even where there is agreement as to the regulatory outcome, policy-makers should not confuse this with agreement on underlying ethical principles. Like many topics concerning the developing genetic and reproductive technologies, cloning remains controversial.

## Competing interests

The author(s) declare that they have no competing interests.

## Authors' contribution

Both authors contributed to the original concept for the paper, the writing and revision of the manuscript and the analysis of the law.

## Pre-publication history

The pre-publication history for this paper can be accessed here:


